# The therapeutic potential of microRNAs to ameliorate spinal cord injury by regulating oligodendrocyte progenitor cells and remyelination

**DOI:** 10.3389/fncel.2024.1404463

**Published:** 2024-05-15

**Authors:** Shanru Qiu, Hui Dai, Yu Wang, Yehua Lv, Bin Yu, Chun Yao

**Affiliations:** Key Laboratory of Neuroregeneration of Jiangsu and Ministry of Education, NMPA Key Laboratory for Research and Evaluation of Tissue Engineering Technology Products, Co-innovation Center of Neuroregeneration, Nantong University, Nantong, China

**Keywords:** microRNA, spinal cord injury, remyelination, oligodendrocyte progenitor cells, oligodendrocyte

## Abstract

Spinal cord injury (SCI) can cause loss of sensory and motor function below the level of injury, posing a serious threat to human health and quality of life. One significant characteristic feature of pathological changes following injury in the nervous system is demyelination, which partially contributes to the long-term deficits in neural function after injury. The remyelination in the central nervous system (CNS) is mainly mediated by oligodendrocyte progenitor cells (OPCs). Numerous complex intracellular signaling and transcriptional factors regulate the differentiation process from OPCs to mature oligodendrocytes (OLs) and myelination. Studies have shown the importance of microRNA (miRNA) in regulating OPC functions. In this review, we focus on the demyelination and remyelination after SCI, and summarize the progress of miRNAs on OPC functions and remyelination, which might provide a potential therapeutic target for SCI treatments.

## 1 Introduction

Spinal cord injury (SCI), one of the most devastating diseases of the central nervous system (CNS), often leads to the permanent loss of sensory and motor functions and imposes huge financial burdens on the families of patients (Ashammakhi et al., 2019). The pathophysiology of traumatic SCI is complex and progressive, including the primary injury (the initial trauma) and the subsequent secondary injury. During the multifactorial secondary injury cascade, a series of biological and cellular events, such as vascular damage, inflammatory cell infiltration, cell apoptosis, and astrocyte activation, can occur and lead to additional damage to the spinal cord (Ahuja et al., 2017; Fan et al., 2022). As the major myelinating cells in the CNS, oligodendrocytes (OLs) exhibit sensitivity to the deteriorated lesion microenvironment after SCI and are lost due to necrosis and apoptosis, leading to demyelination (Almad et al., 2011; Pukos et al., 2019). The improvement of remyelination, during which oligodendrocyte progenitor cells (OPCs) differentiate into mature OLs and re-myelinate axons, may be a promising therapeutic approach to promote functional recovery after SCI (Papastefanaki and Matsas, 2015).

microRNAs (miRNAs) are small non-coding RNAs that can function as gene regulators to participate in multiple biological processes and diseases, which include the physiology and pathology of SCI (Matsuyama and Suzuki, 2019). It has been demonstrated that miRNAs can modulate axon outgrowth, inflammation, angiogenesis, apoptosis, astrogliosis, and remyelination after SCI. This indicates that miRNA may be a potential target for SCI therapy (Ghibaudi et al., 2017, Shi et al., 2017). Here, we will focus on remyelination after SCI and summarize recent advances in miRNAs on OPC functions and remyelination.

## 2 Demyelination and remyelination after SCI

The axon myelin sheaths in the CNS are formed by OLs, which can extend their cytoplasmic membrane to form a tight segmented sheath (Ferreira et al., 2007). The myelin sheaths constantly communicate with axons and provide protection and nutritional support for axons, thus playing an important role in maintaining normal axon function. In addition, myelin can also promote axonal conduction velocity. After SCI, the microenvironment will be severely damaged, resulting in OL necrosis or apoptosis due to ischemia, activation and infiltration of immune cells, and so on (Oyinbo, 2011). The number of OLs in and around the injury site can be reduced to half immediately by one-day post injury (Papastefanaki and Matsas, 2015). The OL apoptosis reaches a peak in only about 1 week and then lasts for at least 3 weeks (Almad et al., 2011). The loss of OLs and demyelination of surviving axons largely lead to the deterioration of neurological function in SCI (Sharp et al., 2010).

**Figure 1:**
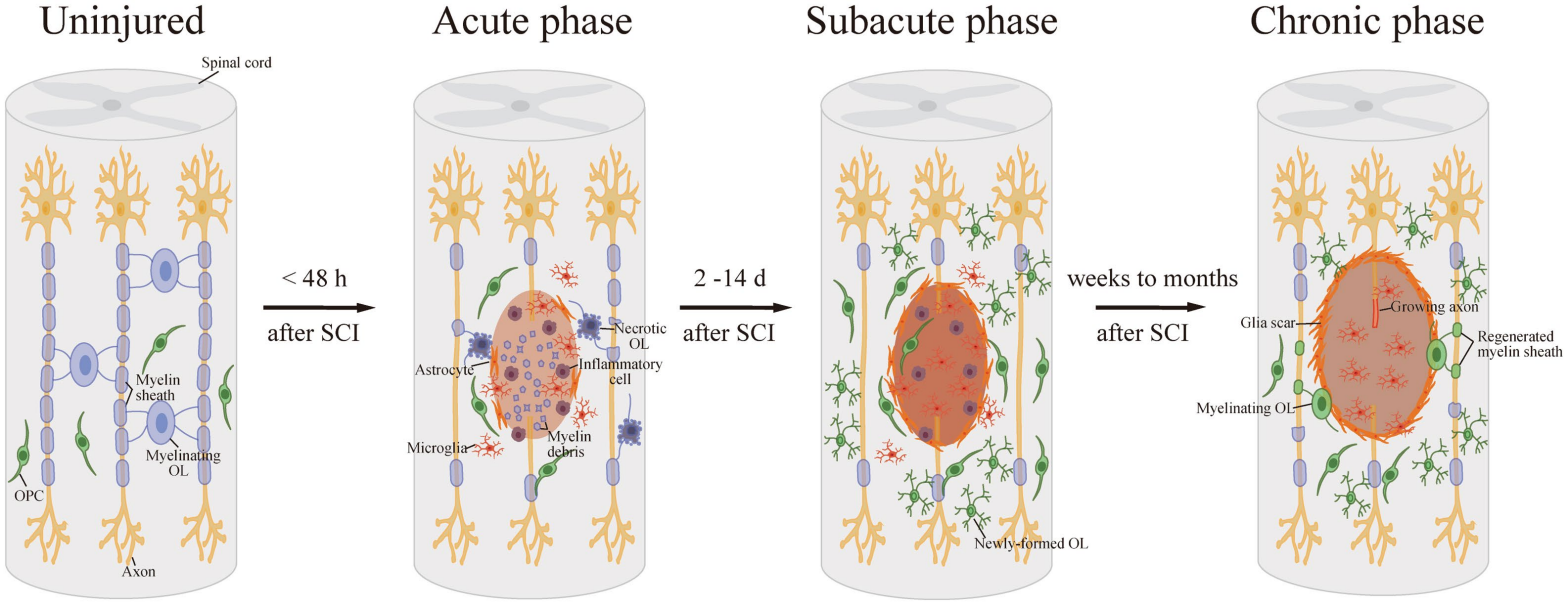
Demyelination and remyelination after SCI. After SCI, OLs are damaged and suffer necrosis or apoptosis, resulting in demyelination. Then, in the subacute phase after SCI, OPCs proliferate strongly and differentiate into newly-formed OLs. Afterward, the new OLs bind to the spare axon and form a detectable dense myelin sheath in the long-term phase.

Meanwhile, OPCs in the lesion area or specifically generated from neural progenitor cells (NPCs) can be activated, proliferate, differentiate into mature OLs, and re-myelinate axons, which compensate for the loss of OLs and demyelination after SCI (Boulanger and Messier, 2014). SCI activates the OPCs around the lesion, which then turn into a bipolar migratory shape and alter gene expression, preparing for the following proliferation and differentiation. Activated OPCs proliferate strongly in the first 3 days after SCI, and continue to grow rapidly for the next 14 days, increasing OPC numbers. The proliferated OPCs migrate to the demyelinated areas and form networks with astrocytes at the edge of the lesion. During this period, OPCs differentiate into newly-formed OLs, which then contact with axons and undergo further differentiation into mature myelinating OLs (Pukos et al., 2019; Uyeda and Muramatsu, 2020). It takes approximately 2 weeks for the new OLs to bind to the spare axon and form a discernible dense myelin sheath after SCI. This spontaneous remyelination can last for at least 6 months post injury, which suggests a long-term dynamic injury microenvironment (Pukos et al., 2023; Figure 1).

However, the endogenous response of OPCs is not enough to offset the massive loss of OLs after injury and can only partially compensate for demyelination with thinner myelin sheaths and shorter internodes (Almad et al., 2011; Duncan et al., 2018). There are currently two main therapeutic strategies for myelin repair following SCI. One approach is to preserve existing OLs during the acute stage of SCI and promote the proliferation and differentiation of endogenous OPCs. For example, Vitamin D (VitD) treatment effectively improved hindlimb movement by rescuing OLs from apoptosis and promoting OPC differentiation and remyelination after SCI (Li et al., 2022). The other approach for remyelination after SCI is to transplant exogenous OPCs or stem cells, such as human embryonic stem cell (ESCs)-derived OPCs, NPCs and fate-restricted neural or glial precursors, which also increase myelinated axons in the lesion and functional improvement (Franklin and Ffrench-Constant, 2008; Manley et al., 2017).

## 3 miRNAs for SCI

miRNAs are short RNAs composed of 20–24 nucleotides without coding potential, but they are capable of suppressing gene expression at post-transcriptional or post-translational levels (Sun et al., 2018). One particular miRNA might have hundreds of target genes and can participate in a variety of biological processes and diseases (Lu and Rothenberg, 2018). miRNA alterations have been observed in different physiological and pathological conditions (Katoh, 2014). There are abundant detectable miRNAs in the brain, many of which are cell-type specific (Cao et al., 2006). For example, miR-124 and miR-128 are mainly expressed in neurons (Nieto-Diaz et al., 2014). Increasing evidence has demonstrated the importance of miRNAs in the CNS. Microarray analyses have been conducted to depict a global miRNA expression pattern during spinal cord development and after injury. Studies have shown that miRNAs can affect a series of processes following SCI, including but not limited to apoptosis, inflammation, neurogenesis, and angiogenesis, all of which have an impact on axon regeneration and functional recovery (Dong et al., 2014, Sun et al., 2018). miR-21, for instance, is a multifunctional miRNA up-regulated after SCI, which is involved in glial scar progression, cell apoptosis, and inflammation. Implantation of a functionalized collagen-I scaffold enriched with miR-21-loaded exosomes immediately after a rat complete T10 spinal cord transection inhibited cell apoptosis, promoted neuron survival, and eventually facilitated SCI repair with a reduced cavity (Liu et al., 2022).

## 4 miRNAs regulate OPC function and remyelination

**Figure 2:**
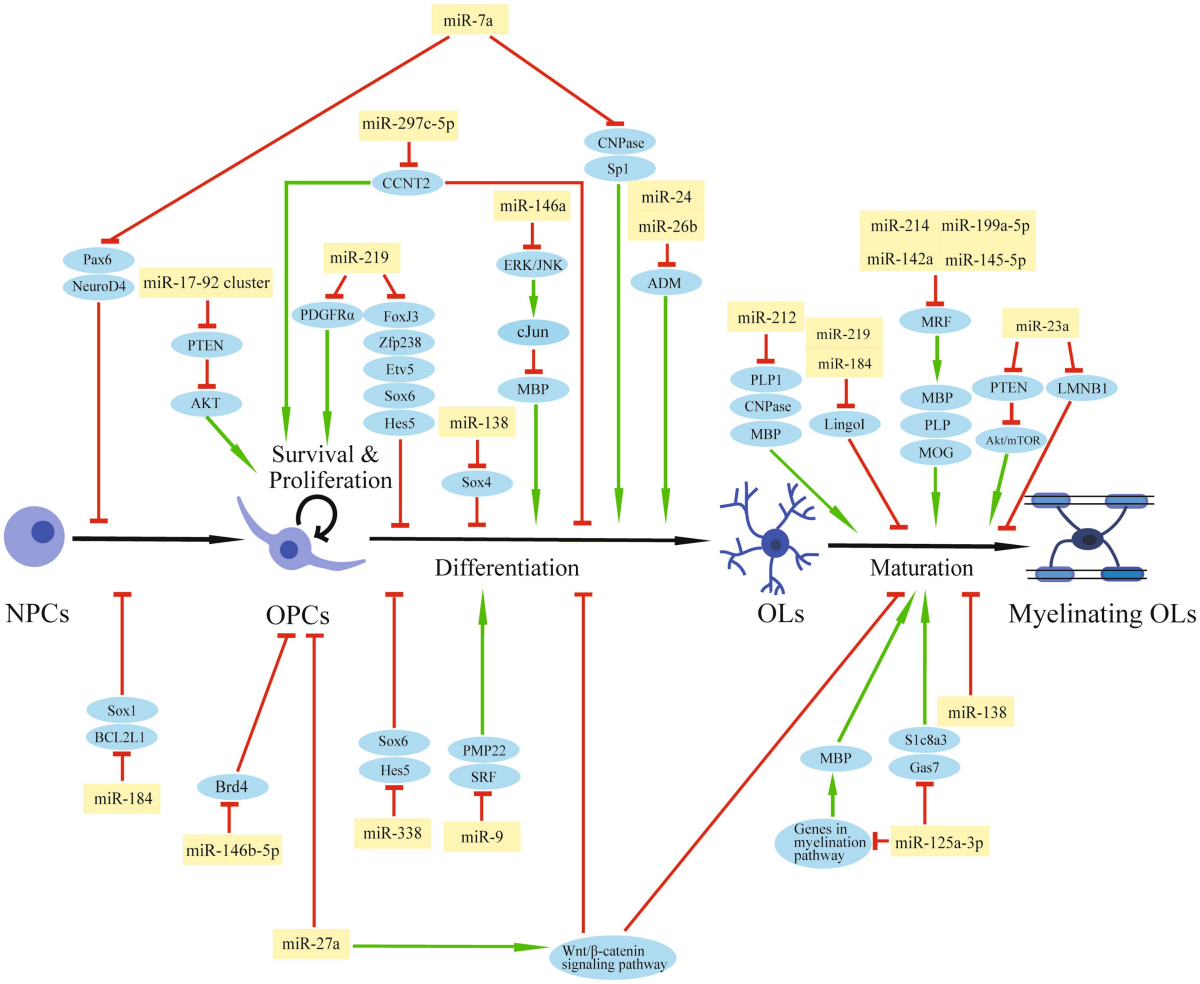
The regulation role of miRNAs in OPCs after SCI. The schematic diagram shows the regulatory network of miRNAs and their target genes that are involved in OPC modulation and myelination.

The Dicer1 enzyme is the key enzyme responsible for the formation of mature miRNAs. The deletion of Dicer1 in OPCs impeded OL differentiation and CNS myelination in mice, suggesting a pivotal role of miRNAs in normal OPC functions and myelin maintenance (Dugas et al., 2010; He et al., 2012). A group of transcription factors, such as SRY-box containing gene (Sox) 5/6/10, zinc finger proteins (Zfp), myelin regulatory factor (MRF), inhibitor of differentiation (Id) Id2/4, and Hes5, are positive or negative regulators of OL differentiation (He et al., 2012). Expressions of essential myelin-associated proteins, such as myelin basic protein (MBP) and proteolipid protein (PLP), are controlled by these transcription factors (Fulton et al., 2010; Aggarwal et al., 2011). Signaling pathways, like Notch or Wnt signaling, participate in OL differentiation and myelination by regulating these transcription factors (Li et al., 2009, Ye et al., 2009, Emery, 2010). Thus, miRNAs that can affect the expression of these differentiation factors may regulate OPC function and the myelination process during CNS development or after injury (Table 1; Figure 2).

**Table 1:** The roles, target genes and experimental models of miRNAs that regulate OPC functions.

miRNA	Function	(Predicted) Gene targets	Experimental model	References
miR-219	Inhibit OPC proliferation Promote OPC differentiation Promote myelination	PDGFRα, FoxJ3, Zfp238, Sox6, Hes5, Etv5, Lingo1	Transgenic mice, LPC-induced demyelination, EAE model, SCI	Li F. et al. (2019); Dugas et al. (2010); Wang H. et al. (2017); Nazari et al. (2021)
miR-338	Promote OPC differentiation	Sox6, Hes5	Transgenic mice, Chick embryos	Dugas et al. (2010); Zhao et al. (2010); Wang H. et al. (2017)
miR-138	Promote OPC differentiation and inhibit OL maturation	Sox4	Transgenic mice	Potzner et al. (2007); Dugas et al. (2010); Yeh et al. (2013)
miR-146a	Promote OPC differentiation	ERK\JNK\c-Jun	Primary rat embryonic OPCs and mouse N20.1 OPCs	Santra et al. (2014)
miR-23a	Promote OL maturation and myelination	PTEN/Akt/mTOR, LMNB1	Transgenic mice, *In vitro* experiments	Lin and Fu (2009); Lin et al. (2013)
miR-184	Promote OPC specification Promote myelination	Sox1, BCL2L1 Lingo1	Mouse embryos	Afrang et al. (2019)
miR-26b	Inhibit OPC differentiation	ADM	SCI	Cheng et al. (2020)
miR-24	Inhibit OPC differentiation	ADM	SCI	Lei et al. (2020)
miR-212	Inhibit OL maturation and myelination	PLP1, CNPase, MBP	SCI	Wang C. Y. et al. (2017)
miR-7a	Promote OPC specification and proliferation, Inhibit OPC differentiation	Pax6, NeuroD4 CNPase, Sp1	NPCs, Mouse embryos	Zhao et al. (2012)
miR-9	Inhibit OPC differentiation	PMP22, SRF	Middle cerebral artery occlusion model	Lau et al. (2008); Buller et al. (2012)
miR-27a	Inhibit OPC proliferation Inhibit myelination	Wnt/β-catenin signaling pathway	P4 mouse pups, Cuprizone-induced demyelination, LPC-induced demyelination	Tripathi et al. (2019)
miR-125a-3p	Inhibit OL maturation	Slc8a3, Gas7, Genes in myelination pathway	LPC-induced demyelination and EAE model	Lecca et al. (2016); Marangon et al. (2020)
miR-199a-5p	Inhibit myelination	MRF	ESC-derived model of OL differentiation, Spinal cord neurotoxicity induced by lidocaine	Letzen et al. (2010); Wang H. et al. (2017)
miR-214	Inhibit myelination	MRF	ESC-derived model of OL differentiation	Letzen et al. (2010)
miR-145	Inhibit myelination Promote OPC proliferation	MRF	Primary rat OPCs	Kornfeld et al. (2021)
miR-142a	Inhibit myelination	MRF	Cuprizone-induced demyelination	Shu et al. (2021)
miR-17-92 cluster	Promote OPC proliferation	PTEN/Akt	Transgenic mice, *In vitro* experiments	Budde et al. (2010)
miR-297c-5p	Inhibit OPC proliferation Promote OPC differentiation	CCNT2	OPC differentiation *in vitro* and callosal development *in vivo*	Kuypers et al. (2016)
miR-146b-5p	Promote OPC survival	Brd4	Oxygen–glucose deprivation injury *in vitro*	Li X. Q. et al. (2019)

By miRNA microarray analysis of rat primary OPCs/OLs, miR-219, miR-338, and miR-138 were identified to be significantly induced during the transition from OPCs to OLs (Dugas et al., 2010). miR-219 is the most strongly expressed miRNA in OLs among these three miRNAs and exhibits a great potential to promote differentiation by reducing the expression of genes that suppress differentiation, such as forkhead box J3 (FoxJ3), ZFP238, Sox6, Hes5, and Etv5. At the same time, miR-219 can inhibit OPC proliferation by targeting platelet-derived growth factor receptor alpha (PDGFRα; Stolt et al., 2006; Zhao et al., 2010; Wang H, et al., 2017). Additionally, miR-219 mimics enhanced remyelination in demyelinating injury models by inhibiting leucine-rich repeat and Ig domain-containing 1 (Lingo1), an OL myelination inhibitor (Wang H. et al., 2017). A number of experiments have been conducted to confirm the repair function of miR-219 for SCI. Administration of miR-219 agomiR in rats after unilateral C5 contusion SCI significantly promoted OPC differentiation, increased OL number, enhanced myelin repair, and partially improved the forelimb motor function (Li F. et al., 2019). OPCs overexpressed with miR-219 were transplanted into rats after T12 compression SCI within 1 week, and a higher OL differentiation rate was observed 8 weeks after the transplantation, with enhanced white matter and increased myelin area after injury. Also, transplantation of miR-219-overexpressed OPCs increased the number of regenerated axons and promoted functional recovery (Nazari et al., 2021). miR-338 also facilitates OPC differentiation by targeting Sox6 and Hes5 (Zhao et al., 2010). Mice deleted with both miR-338 and miR-219 have a more severe dysmyelination phenotype than miR-219 mutation mice, indicating that miR-338 has a synergistic effect with miR-219 on OL differentiation (Wang H, et al., 2017). Unlike miR-219, which induces OL differentiation both in the early and late stages, miR-138 promotes the early phase of OL differentiation, probably by targeting the OL maturation repressor Sox4 (Potzner et al., 2007; Yeh et al., 2013). On the other hand, miR-138 might impede the later differentiation progression and prolong the OL immature stage (Dugas et al., 2010).
In primary OPCs or OPC cell lines, miR-146a can be induced by the multipotent peptide Thymosin 4 (Tβ4). This up-regulated miRNA can then promote OPC differentiation by suppressing the activation of ERK/JNK1 and the MBP synthesis repressor c-Jun (Santra et al., 2014).

miR-23a is up-regulated in the injured area of multiple sclerosis (MS; Junker et al., 2009). The miR-23a transgenic mice showed increased OL differentiation and enhanced myelination in the CNS. Mechanism study indicated that miR-23a might target PTEN and activate the Akt/mTOR pathway, which promotes myelin gene expression (Lin et al., 2013). In addition, miR-23 can also promote myelination by inhibiting lamin B1 (LMNB1), a negative regulator of OL differentiation which can lead to premature arrest of OL maturation (Lin and Fu, 2009).

It has been reported that miR-184 is sharply increased during the differentiation phase of glia-restrictive precursors to OL precursors, and is one of the most up-regulated miRNAs in the final OL transition stage. miR-184 overexpression in NPCs can effectively stimulate the differentiation of OL lines by repressing Sox1 and BCL2 Like 1 (BCL2L1), which are inducers for neural and astrocyte lineage commitment. In addition, miR-184 can inhibit the expression of Lingo1, which is a negative regulator for the differentiation and myelination of OLs (Afrang et al., 2019).

After rat T10 spinal cord contusion, miR-26b decreased in a time-dependent manner, and miR-26b agomiR treatment significantly reduced the BBB score from day 7 to 28 post-injury, indicating that miR-26b can aggravate motor dysfunction after SCI. Further studies showed that miR-26b exerted this negative effect by inhibiting adrenomedullin (ADM) and OPC differentiation (Cheng et al., 2020). Also, the up-regulation of miR-24 after SCI can reduce ADM and MBP expression, inhibit OPC differentiation, and increase inflammatory factor expression (Lei et al., 2020).

miR-212 is decreased at the lesion site after contusion SCI in rats, especially in OLs. Overexpression of miR-212 in OPCs reduced the OL typically expressed proteins, like 2′, 3′-cyclic nucleotide 3′-phosphodiesterase (CNPase), PLP1, and MBP, suggesting a suppressive effect of miR-212 on OL maturation. The decline of miR-212 in OLs after SCI may promote the myelination ability of surviving OLs or the transition from endogenous OPCs to mature OLs in the spinal cord (Wang C Y, et al., 2017).

In NPCs or mouse embryos, overexpression of miR-7a, a highly enriched miRNA in OPCs, accelerated OL lineage cell generation and kept the precursor stage of these cells. Mechanically, miR-7a directly inhibited pre-neuronal differentiation factors such as paired box 6 (Pax6) and neurogenic differentiation 4 (NeuroD4), and myelin-related genes, such as CNPase and Sp1 (Zhao et al., 2012).

A miRNA microarray analysis of OL lineage cells obtained from postnatal rat brains identified miR-9 with decreased expression from OPC to OL differentiation. Peripheral myelin protein 22 (PMP22) was the predicted target of miR-9 during OL differentiation (Lau et al., 2008). Additionally, miR-9 disturbs OPC differentiation by targeting serum response factor (SRF), which facilitates OL generation (Buller et al., 2012).

miR-27a was elevated in demyelinating diseases, such as MS. The increased miR-27a can lead to OPC cell-cycle arrest and inhibit OPC proliferation. Also, miR-27a may activate the Wnt/β-catenin signaling pathway, a canonical pathway for OL development, by inhibiting adenomatous polyposis coli (APC) level, thereby impeding OPC differentiation and myelination. miR-27a administration in P4 mouse pups or lysolecithin (LPC)-induced demyelinating mice significantly reduced myelin sheaths and mature OLs, indicating a crucial role for OL-specific miR-27a in myelination during development and remyelination after injury (Tripathi et al., 2019).

Another miRNA, miR-125a-3p, was also abnormally elevated in MS patients and the spinal cord of EAE mice. It is supposed that miR-125a-3p might hinder the repairment of demyelinating lesions and exacerbate the development of MS by blocking OPC differentiation (Lecca et al., 2016). In an LPC-induced demyelinating model, overexpression of miR-125a-3p inhibited OL maturation, while silencing miR-125a-3p promoted post-injury myelin repair. This effect on OL maturation might be mediated by the direct interaction of miR-125a-3p with the sodium-calcium membrane transporter Slc8a3, which encodes a transmembrane Na^+^/Ca^2+^ exchanger involved in OPC differentiation and myelin synthesis. Gas7, a growth-arrest protein, was also a potential miR-125a-3p target gene during OL maturation (Marangon et al., 2020). Furthermore, miR-125a-3p might affect MBP expression by genes regulating myelination, such as Fyn, Nrg1, RhoA, p38, and Smad4 (Lecca et al., 2016). Overall, these studies indicated that miR-125a-3p synergistically inhibited OL differentiation and maturation in different mechanisms.

In ESCs-derived OL differentiation, miR-199a-5p was dysregulated and targeted C11Orf9 (a homologous gene of MRF), which is an essential regulator for OL maturation and CNS myelination (Emery et al., 2009, Letzen et al., 2010). In a lidocaine-induced neurotoxicity model, the treatment of miR-199a-5p antagomiR alleviated the sensory disturbance and myelin damage by reversing the significant reduction of myelin regulatory factor MRF and its downstream factors, such as MBP, PLP, and MOG. Thus, inhibition of miR-199a-5p promoted remyelination and facilitated myelin repair (Zhang Z, et al., 2021). Also, miR-214, miR-145-5p, and miR-142a are negative regulators of OPC differentiation and myelination in the CNS by targeting MRF during OL development or after injury (Letzen et al., 2010, Kornfeld et al., 2021; Shu et al., 2021).

In OL lineage cells, the miR-17-92 cluster is enriched (Budde et al., 2010; de Faria et al., 2012). Mice with miR-17-92 deletion showed a significant reduction in OL number, indicating the pivotal role of this miRNA cluster in OL development. Further *in vitro* experiments illustrated that miR-17-92 promoted cell proliferation by reducing the tumor suppressor gene PTEN and activating downstream Akt signaling (Budde et al., 2010). Besides the miR-17-92 miRNA cluster, the above-mentioned miR-7a and miR-145 can also promote OPC proliferation (Zhao et al., 2012; Kornfeld et al., 2021).

There was an up-regulated expression of miR-297c-5p during OPC differentiation *in vitro* and development *in vivo*. Overexpression of miR-297c-5p on OPCs disrupted the transition from G1/G0 to S-phase, suggesting an inhibitory role of miR-297c-5p on the cell cycle. Furthermore, miR-297c-5p can block the expression of the differentiation inhibitor cyclin T2 (CCNT2) and enhance mature OL numbers (Kuypers et al., 2016).

miR-146b-5p was decreased in OPCs in an *in vitro* oxygen–glucose deprivation-induced injury model. miR-146b-5p mimic transfection increased the growth and viability of the damaged OPCs by reducing apoptosis and oxidative stress. Mechanically, miR-146b-5p decreased the expression of bromodomain-containing protein 4 (Brd4) and activated the antioxidant system (Li X. Q. et al., 2019).

## 5 Outlook

SCI is a complex pathophysiological process. miRNAs can modulate OPC functions and enhance remyelination after injury, thereby providing a potential therapeutic target for facilitating SCI repair and functional recovery. However, clinical translation and application of miRNA-based therapies are rare, especially for SCI. miRNA-based therapies face numerous challenges, including delivery, stability, and immune responses (Hydbring and Badalian-Very, 2013, Sun et al., 2018). Exposed miRNAs are difficult to be absorbed by cells due to their negative charge, off-target effect, short half-life in the systemic circulation, and rapid degradation or inactivation by abundant nucleases in the bloodstream (Silvestro and Mazzon, 2022).

Several delivery methods have been developed to improve the stability and tissue penetration of miRNAs *in vivo* (Dasgupta and Chatterjee, 2021). Lentivirus and recombinant adeno-associated viruses have been used to deliver miRNAs in the spinal cord for SCI treatment (Theis et al., 2017; Zhang H, et al., 2021). However, the packaging capacity of viral vectors is limited due to size limitations, and it takes time for viral-mediated intervention (Sun et al., 2018). In the past few years, growing studies have promoted SCI repair with miRNAs through non-viral delivery methods, such as exosomes or biomaterial-based nanoparticles (Chakraborty et al., 2021; Feng et al., 2021; Shen and Cai, 2023). Exosomes can pass through the blood–brain barrier according to their lipid bilayer structure, and they can protect their loads from enzyme decomposition or other processes, ensuring the safety and stability of the cargo (Ha et al., 2016). For example, the miR-709 delivered by exosomes can significantly reduce the inflammatory response, attenuate microglia pyroptosis, and improve motor function remodeling after contusion SCI in mice (Xiong et al., 2022). On the other hand, biomaterial-based engineered nanoparticles can also cross the blood–brain barrier, realize target delivery of miRNAs to certain organs and cells, and sustain the release of miRNAs (Lee et al., 2019). miR-124-3p-loaded nanoparticles coated by a modified stem cell membrane could cross the damaged BSCB, accumulate in the lesion area, and release sufficient miRNAs to modulate the local microenvironment and promote axon outgrowth after SCI (Fan et al., 2024). In addition, nanoparticles can be modified with certain peptides or antibodies to target specific cells in the lesion microenvironment. As for OPCs, it was reported that lipid nanoparticles conjugated with anti-CD140 mAb could specifically target PDGFRα/CD140a OPCs (Xu et al., 2024).

In general, in this review, we focus on remyelination after SCI and summarize miRNAs that can regulate OPC functions, which might be used for SCI treatments. Further studies are needed to explore more functional miRNAs for remyelination after SCI and develop efficient delivery methods for miRNA-based therapies.

## Author contributions

SQ: Funding acquisition, Data curation, Writing – original draft. HD: Data curation, Writing – original draft. YW: Writing – original draft. YL: Writing – original draft. BY: Supervision, Writing – review & editing. CY: Supervision, Writing – review & editing, Conceptualization, Funding acquisition.
